# Supplementary material for: Discovering cryptic splice mutations in cancers via a deep neural network framework
Source: NAR Cancer. 2023 Mar 15;5(2):zcad014. doi: 10.1093/narcan/zcad014 (PMC10015341; doi:10.1093/narcan/zcad014)
Supplement: zcad014_Supplemental_Files [file zcad014_supplemental_files.zip › Revised_Supplementary Data.pdf]

Supplementary Data

Discovering cryptic splice mutations in cancers via a deep neural network framework

Teboul *et el.*

List of Supplementary Figures

Supplementary Figure S1: Validation scheme for predicted splice-impacting mutations.
Supplementary Figure S2: Proportion of splicing alteration types and VEP annotations according to SpliceAI DS scores
Supplementary Figure S3: MutSigCV q-values including or not cryptic splice mutations for each HMF and TCGA series

List of Supplementary Tables (provided as Excel files)

Supplementary Table S1: Cryptic splice mutations identified by SpliceAI, MaxEntScan and GeneSplicer in the LICA-FR series.
Supplementary Table S2: Validation of 3,000 mutations predicted to impact splicing in the TCGA series
Supplementary Table S3: Extra-significant genes (identified by MutSigCV when including cryptic splice mutations) that belong to the Cancer Gene Census
Supplementary Table S4: Extra-significant genes (identified by MutSigCV when including cryptic splice mutations) that do not belong to the Cancer Gene Census
Supplementary Table S5: Mutation rates of Cancer Gene Census genes in a large pan-cancer dataset
Supplementary Table S6: Mutational signatures contributing preferentially to splice mutations.

A

$$RUNJ = \frac{\#M_a}{\#M_a + \#M_n} - \frac{\#C_a}{\#C_a + \#C_n}$$

$\#M_a$: number of abnormal reads in the mutated sample

$\#M_n$: number of normal reads in the mutated sample

$\#C_a$: number of abnormal reads in the control samples (PON or POT)

$\#C_n$: number of normal reads in the control samples (PON or POT)

B

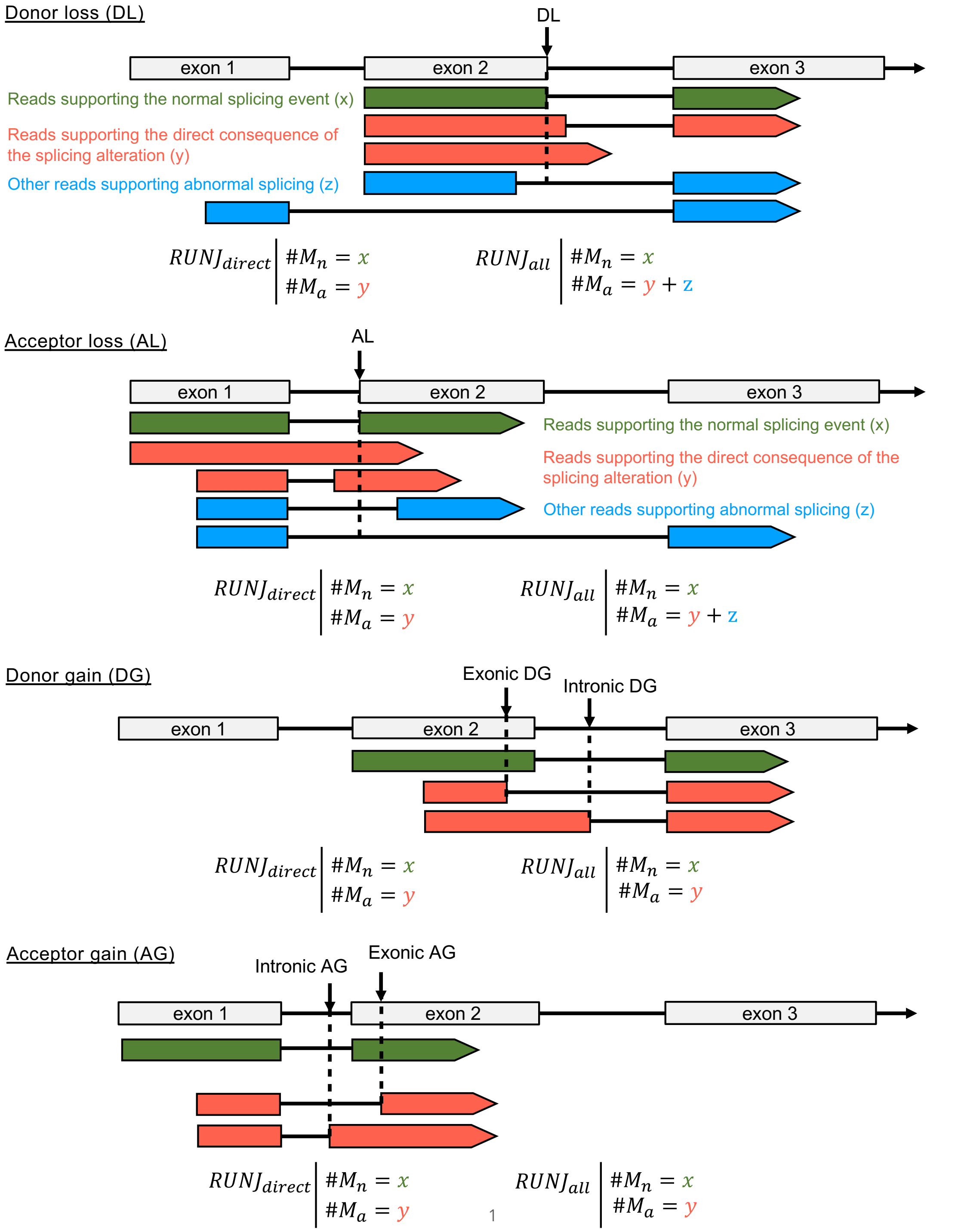

Supplementary Figure S1: Validation scheme for predicted splice-impacting mutations. A Formula of the Relative Usage of the Novel Junction (RUNJ) score, comparing the proportion of reads supporting abnormal splicing in the mutated sample against a set of control samples. RUNJ scores are computed against a Panel Of Normal samples (PON) and against a Panel Of

Tumors (POT). Both scores need to meet the required threshold (0.01) to ensure that the abnormal splicing is specific to the mutated tumor. **B** The number of mutated reads is computed in two ways The $RUNJ_{direct}$ score considers only abnormal reads that support the most direct consequence of the splicing alteration (in red), e.g. reads passing trough the essential donor for donor loss events). However, the observed consequence on the RNA can be different from the expected one. For example, a donor loss could favor usage of a cryptic donor site upstream within the exon, leading to a truncated exon or even full exon skipping (in blue). In that case, we would not detect RNA-seq reads continuing from the exon to the intron, but rather abnormal junctions linking the cryptic donor to the next acceptor site. The $RUNJ_{all}$ score accounts for these situations by considering all abnormal reads at the considered exon-intron boundary. This panel represents, for each splicing mutation category, reads supporting the normal splicing event (in green), those supporting the direct consequence of the splicing alteration (in red) and other reads supporting abnormal splicing (in blue). Reads taken into account to compute the number of normal and abnormal reads in the $RUNJ_{direct}$ and $RUNJ_{all}$ are shown for each mutation category. Note that for donor and acceptor gains, the two scores are equivalent since a single consequence is expected from the splicing mutation.

A

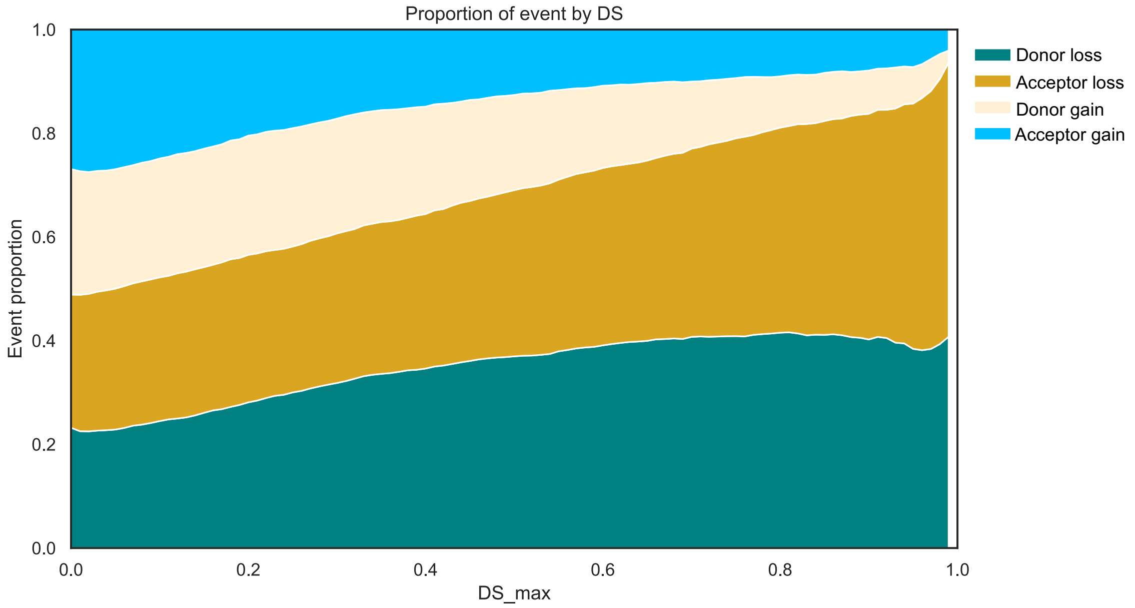

B

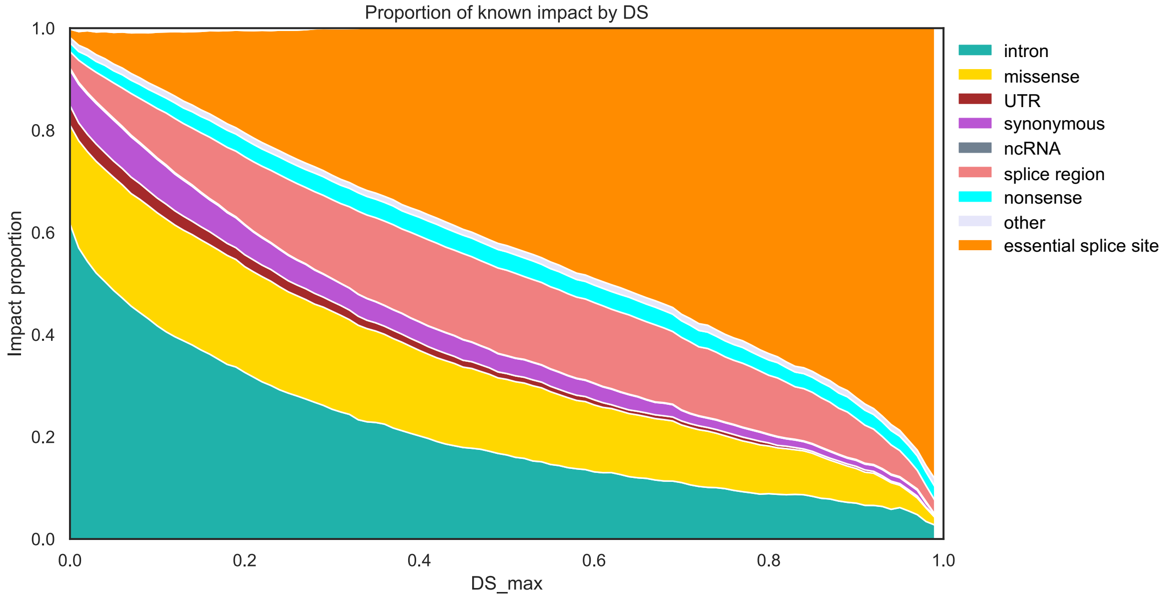

Supplementary Figure S2: Proportion of splicing alteration types and VEP annotations according to SpliceAI DS scores. A Proportion of donor loss (DL), acceptor loss (AL), donor gain (DG) and acceptor gain (AG) events as a function of SpliceAI delta score (DS). **B** Proportion of mutation categories (annotated by the Variant Effect Predictor) as a function of SpliceAI delta score (DS).

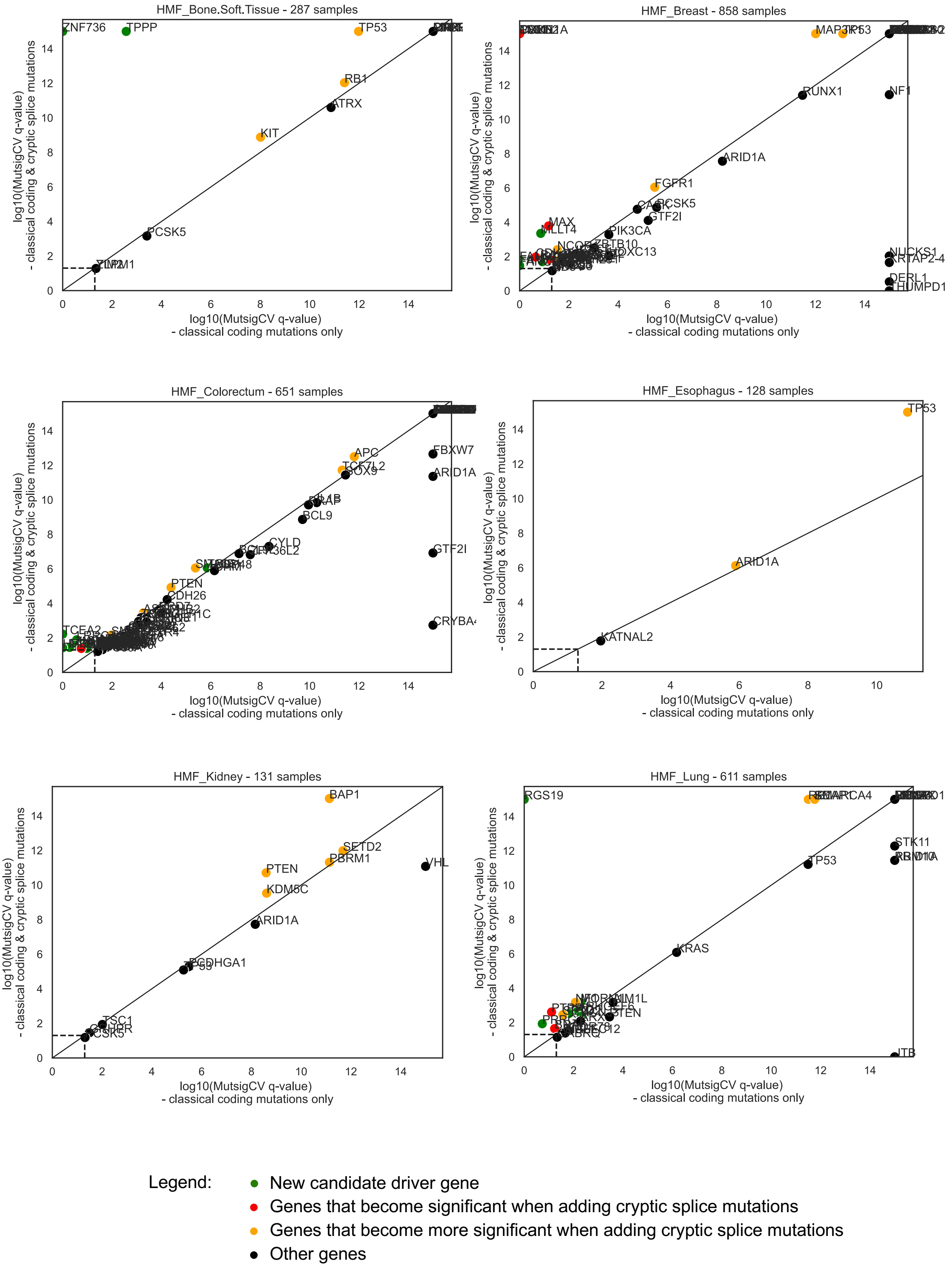
HMF_Bone.Soft.Tissue - 287 samples
ZNF736
TPPP
TP53
RB1
ATRX
KIT
PCSK5
HMF_Breast - 858 samples
MAP3K1
TP53
RUNX1
NF1
ARID1A
FGFR1
PCSK5
GTF2I
MAX
MLLT4
PIK3CA
ZBTB10
HOXC13
NUCKS1
KRTAP2-4
DERL1
THUMPD1
HMF_Colorectum - 651 samples
APC
TCF7L2
SOX9
FBXW7
ARID1A
BCL9
CYLD
GTF2I
PTEN
CDH26
TCEA2
CRYBA4
HMF_Esophagus - 128 samples
TP53
ARID1A
KATNAL2
HMF_Kidney - 131 samples
BAP1
SETD2
PBRM1
VHL
PTEN
KDM5C
ARID1A
PCDHGA1
TSC1
PCSK5
HMF_Lung - 611 samples
RGS19
SMARCA4
STK11
TP53
KRAS
PTEN
JTB
log10(MutsigCV q-value)
- classical coding mutations only
log10(MutsigCV q-value)
- classical coding & cryptic splice mutations
Legend:
New candidate driver gene
Genes that become significant when adding cryptic splice mutations
Genes that become more significant when adding cryptic splice mutations
Other genes

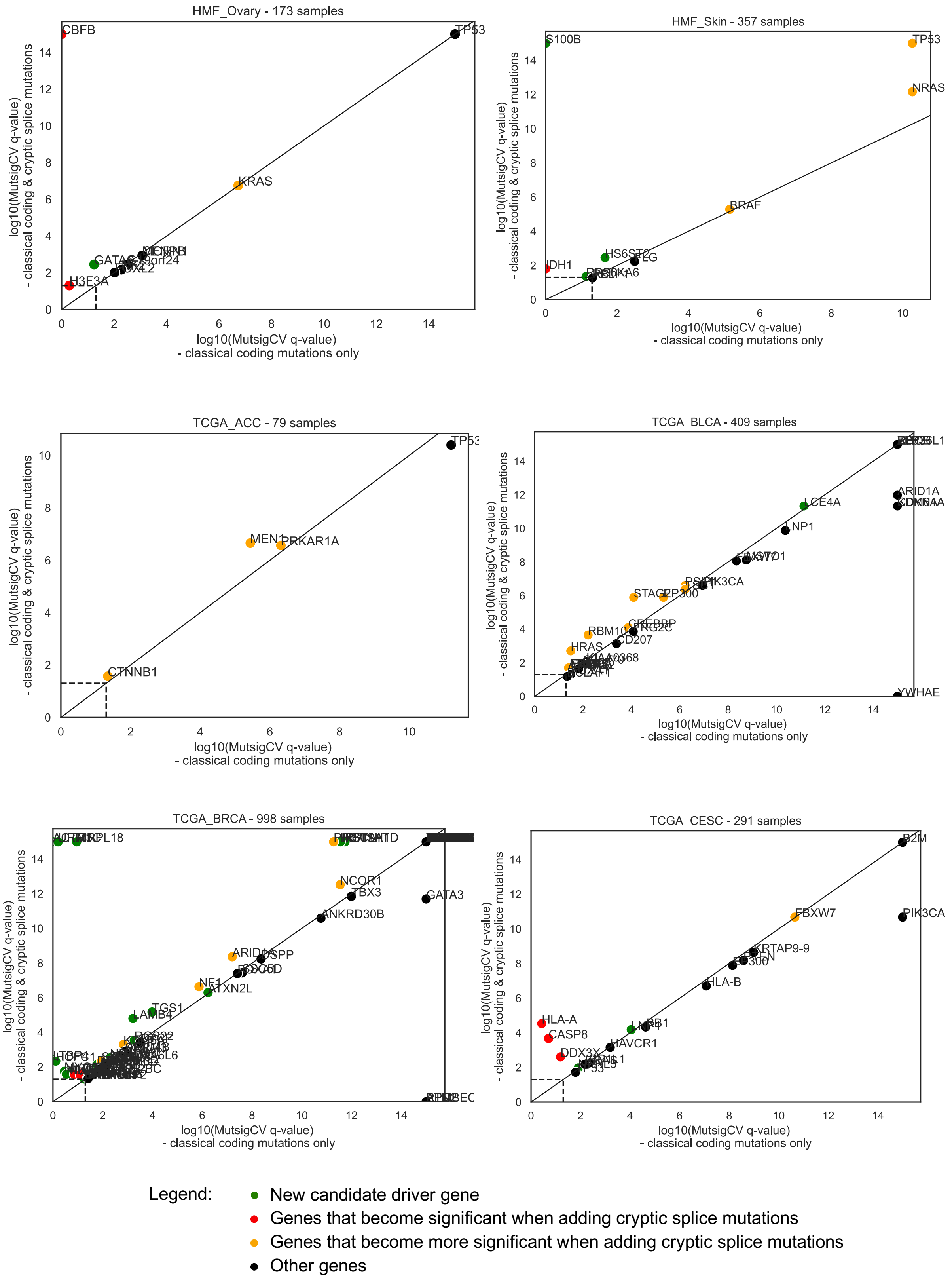

HMF_Ovary - 173 samples
CBFB
TP53
KRAS
H3F3A
log10(MutsigCV q-value)
- classical coding & cryptic splice mutations
log10(MutsigCV q-value)
- classical coding mutations only
HMF_Skin - 357 samples
S100B
TP53
NRAS
BRAF
HS6ST2
IDH1
TCGA_ACC - 79 samples
MEN1
PRKAR1A
CTNNB1
TCGA_BLCA - 409 samples
ARID1A
LCE4A
LNP1
PIK3CA
STAG2
EP300
CREBBP
RBM10
CD207
HRAS
YWHAE
TCGA_BRCA - 998 samples
NCOR1
TBX3
GATA3
ANKRD30B
ARID1A
NF1
ATXN2L
TGS1
LAMB4
TCGA_CESC - 291 samples
B2M
PIK3CA
FBXW7
KRTAP9-9
EP300
HLA-B
HLA-A
CASP8
HAVCR1
Legend:
New candidate driver gene
Genes that become significant when adding cryptic splice mutations
Genes that become more significant when adding cryptic splice mutations
Other genes

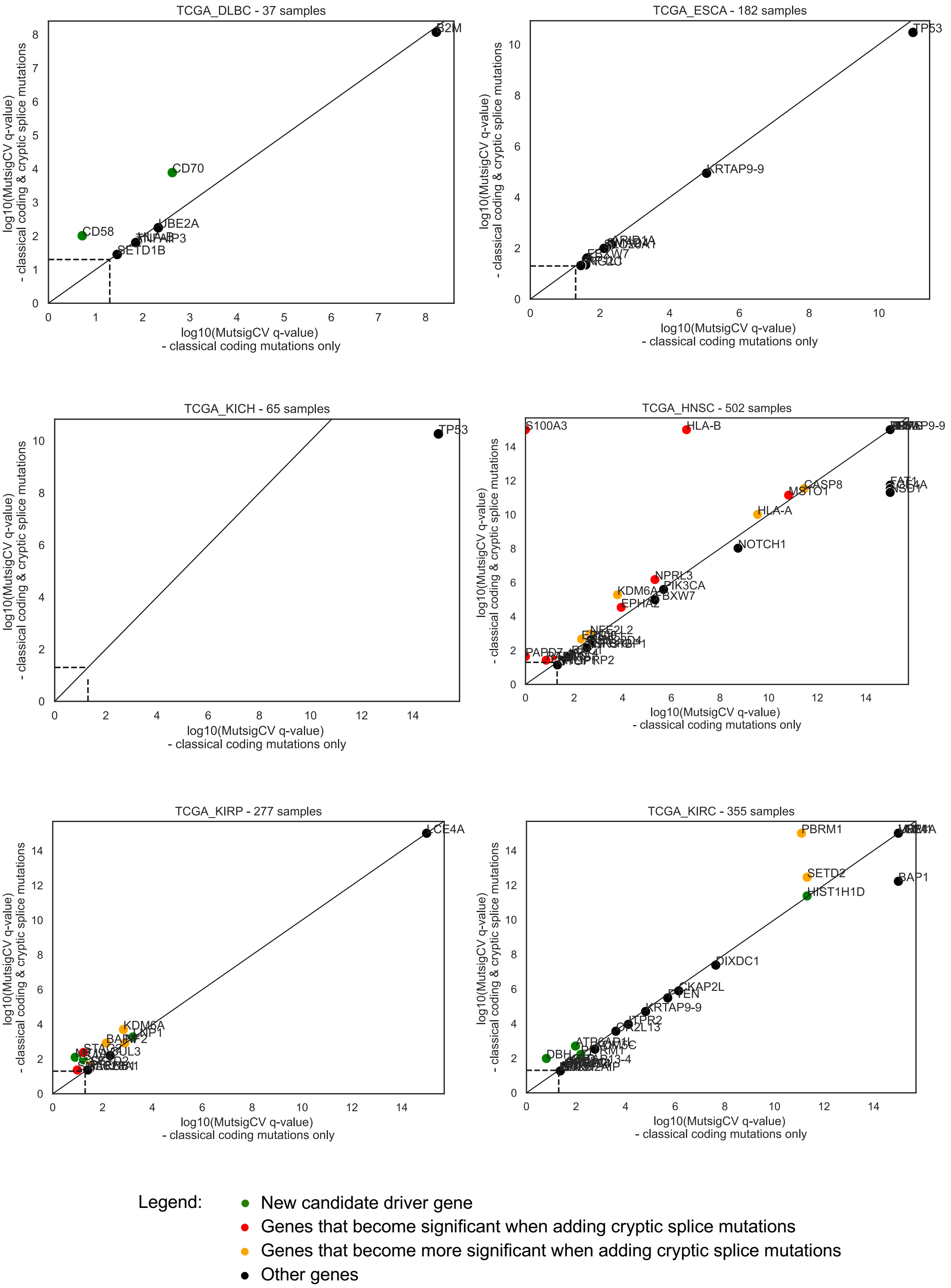
TCGA_DLBC - 37 samples
B2M
CD70
CD58
UBE2A
SETD1B
TCGA_ESCA - 182 samples
TP53
KRTAP9-9
TCGA_KICH - 65 samples
TP53
TCGA_HNSC - 502 samples
S100A3
HLA-B
CASP8
HLA-A
NOTCH1
NPRL3
PIK3CA
KDM6A
EPHA2
NFE2L2
TCGA_KIRP - 277 samples
LCE4A
KDM6A
TCGA_KIRC - 355 samples
PBRM1
SETD2
BAP1
HIST1H1D
DIXDC1
CKAP2L
PTEN
KRTAP9-9
ITPR2
DBH
log10(MutsigCV q-value)
- classical coding & cryptic splice mutations
log10(MutsigCV q-value)
- classical coding mutations only
Legend:
New candidate driver gene
Genes that become significant when adding cryptic splice mutations
Genes that become more significant when adding cryptic splice mutations
Other genes

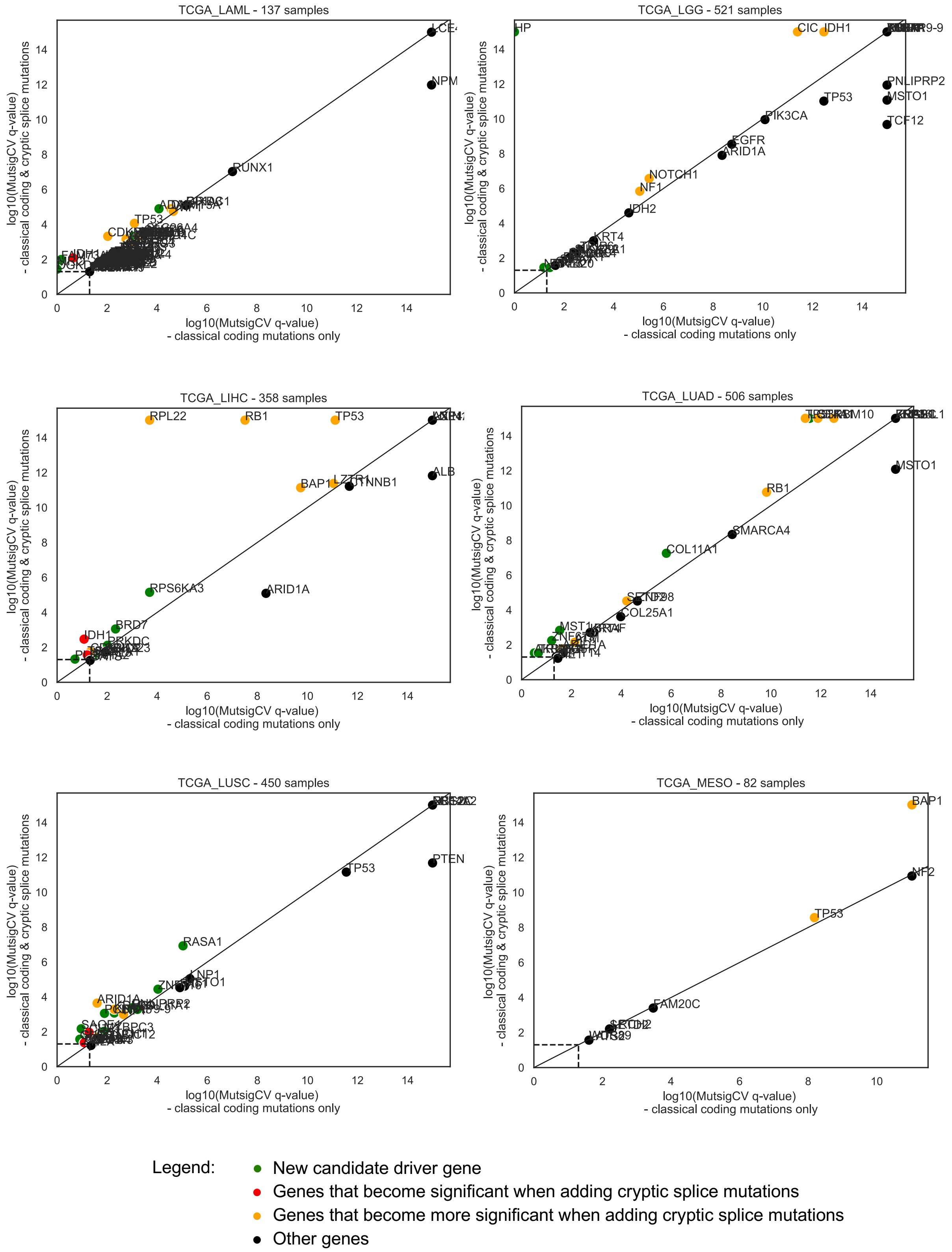

TCGA_LAML - 137 samples
TCGA_LGG - 521 samples
TCGA_LIHC - 358 samples
TCGA_LUAD - 506 samples
TCGA_LUSC - 450 samples
TCGA_MESO - 82 samples
log10(MutsigCV q-value)
- classical coding mutations only
log10(MutsigCV q-value)
- classical coding & cryptic splice mutations
RUNX1
TP53
CIC
IDH1
PNLIPRP2
MSTO1
TCF12
PIK3CA
EGFR
ARID1A
NOTCH1
NF1
IDH2
KRT4
RPL22
RB1
BAP1
ALB
RPS6KA3
BRD7
MSTO1
SMARCA4
COL11A1
COL25A1
PTEN
RASA1
NF2
FAM20C
Legend:
New candidate driver gene
Genes that become significant when adding cryptic splice mutations
Genes that become more significant when adding cryptic splice mutations
Other genes

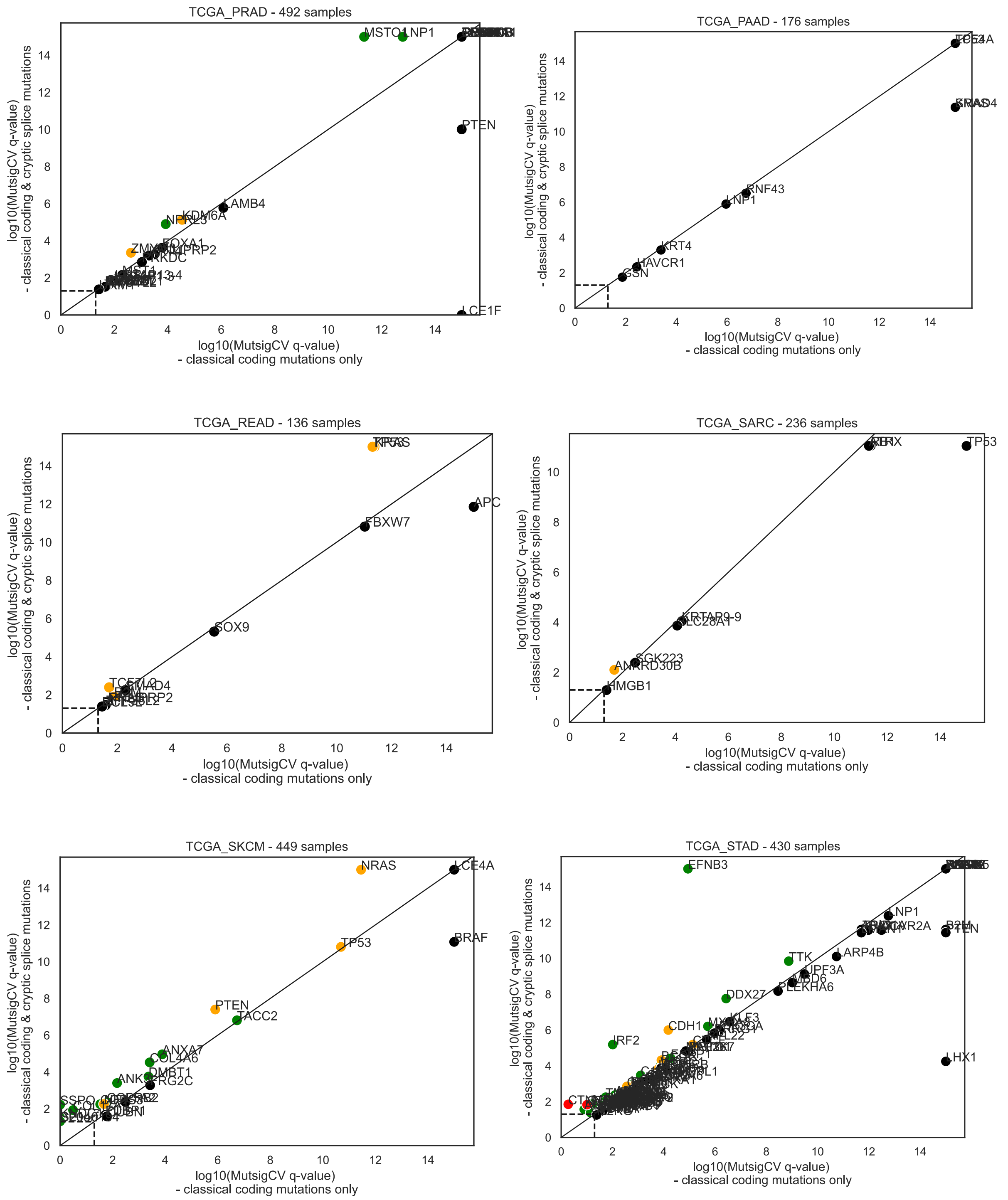

Legend:
- New candidate driver gene
- Genes that become significant when adding cryptic splice mutations
- Genes that become more significant when adding cryptic splice mutations
- Other genes

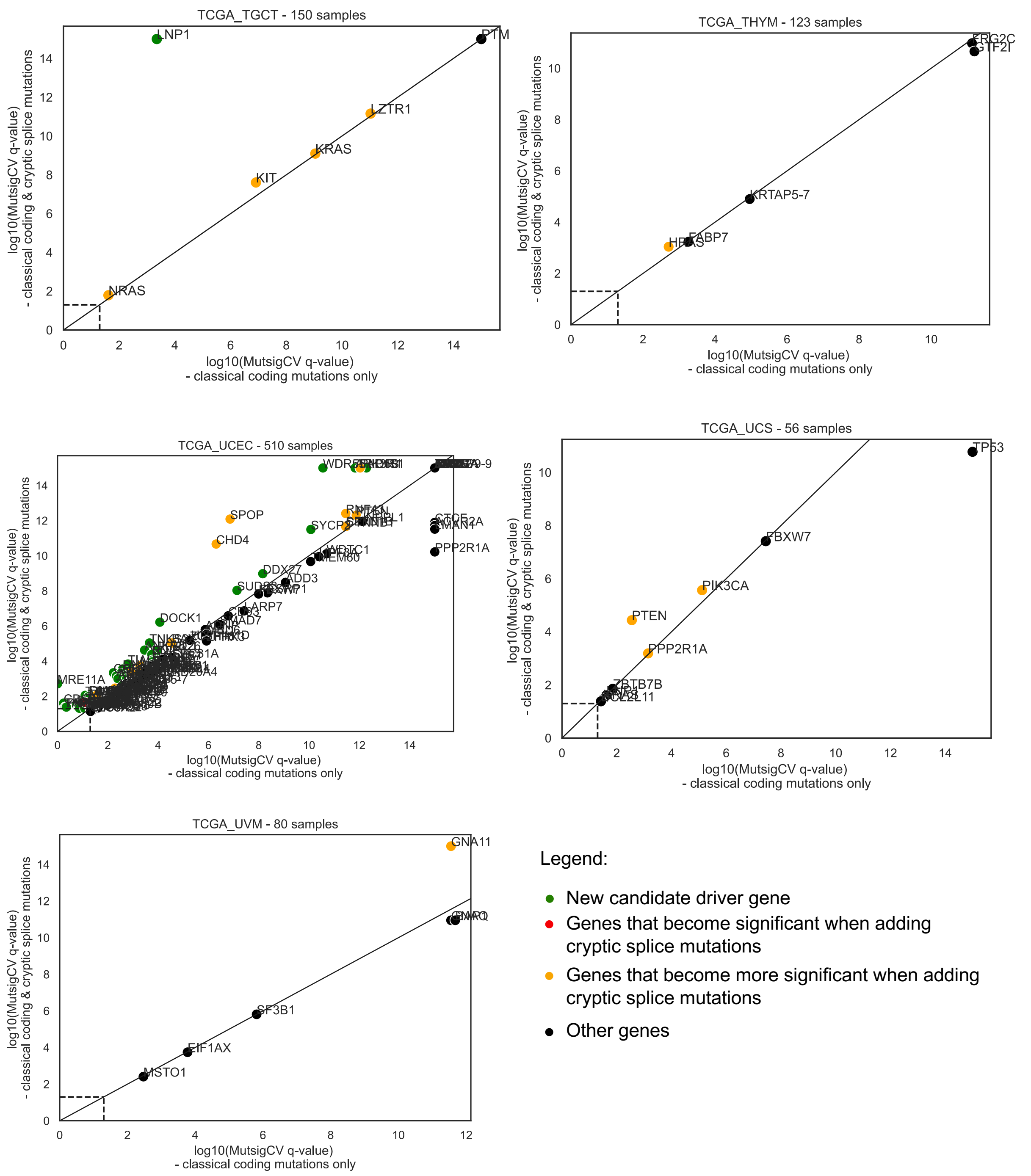

Supplementary Figure S3: MutSigCV q-values including or not cryptic splice mutations for each HMF and TCGA series. MutSigCV q-values were calculated for each HMF/TCGA series considering only classical coding and essential splice mutations, or when adding cryptic splice mutations. Genes significant in one or the other test are represented with a color code indicating whether they become significant or increase their significance level when adding cryptic splice mutations.
